# A consensus definition of creativity in surgery: A Delphi study protocol

**DOI:** 10.1371/journal.pone.0314445

**Published:** 2024-12-05

**Authors:** Alex Thabane, Tyler McKechnie, Phillip Staibano, Vikram Arora, Goran Calic, Jason W. Busse, Sameer Parpia, Mohit Bhandari

**Affiliations:** 1 Department of Health Research Methods, Evidence, and Impact, McMaster University, Hamilton, Ontario, Canada; 2 Department of Surgery, McMaster University Medical Center, Hamilton, Ontario, Canada; 3 DeGroote School of Business, McMaster University, Hamilton, Ontario, Canada; 4 Department of Anesthesia, McMaster University Medical Center, Hamilton, Ontario, Canada; Beatrix Children’s Hospital, University Medical Center Groningen, NETHERLANDS, KINGDOM OF THE

## Abstract

**Introduction:**

Clear definitions are essential in science, particularly in the study of abstract phenomena like creativity. Due to its inherent complexity and domain-specific nature, the study of creativity has been complicated, as evidenced by the various definitions used to describe it and the multitude of tools which claim to measure it. Surgery is a safety-critical profession where creativity could be useful in navigating unforeseen problems and circumstances, as well as developing new innovations to improve patient outcomes. To validly and reliably study creativity in surgery, a surgery-specific definition is required. We aim to develop a consensus definition of creativity in surgery, utilizing the existing creativity literature and surgeon input.

**Methods and analysis:**

The objective of this study is to generate a consensus definition of creativity in surgery. We will first conduct a focus group comprised of 4–12 highly experienced surgeons to generate knowledge on surgeons’ perceptions, attitudes and beliefs about creativity in surgery, collect real-world examples of creativity in surgery, and obtain opinions on the existing definitions of creativity in the literature. The selection of focus group participants will be performed using purposive sampling of the chairs and/or chiefs of each surgical sub-specialty at our home institution. Several questions relating to creativity in surgery will be posed to the focus group, to be rated using a 7-point Likert scale and used as prompts for group discussion. We will also search MEDLINE, PsycINFO and EMBASE to find definitions of creativity in the scientific literature. Six definitions, chosen based on citation frequency and relevancy to surgery, will be presented to the focus group for ranking and discussion. Lastly, in addition to novelty and effectiveness, which are widely accepted as necessary components of creativity, the focus group will be asked to consider the necessity of other components for creativity in surgery, sourced from the scientific literature. Descriptive and thematic analyses are planned for the quantitative and qualitative data, respectively. The results of the focus group will be incorporated in the drafting of five definitions of creativity in surgery, which will be presented as initial Delphi statements in the Delphi study. For the Delphi panel, we will perform non-probability purposive sampling of surgeons and surgeon trainees at our home institution, with a minimum panel size of 12. Panellists will be asked to select the definition of creativity most relevant to surgery, with each Delphi round electronically delivered. After each round, the steering committee will meet to review the results and adjust the statements for the next round based on the feedback. A maximum of 5 rounds will be performed, or until consensus is reached (≥75% agreement). Recruitment is scheduled to begin on 1 August 2024.

**Ethics and dissemination:**

All focus group and panellists will be given written and verbal information on the study and provide signed, informed consent. We plan to publish the results of our study in a creativity science- or surgery-focused journal to disseminate the results of our study to relevant stakeholders. We also plan to present the results of our research at local, national, and international conferences.

## Introduction

Clear definitions are essential in science, particularly in the study of abstract phenomena: they facilitate the replication of studies and provide the conceptual foundation that directs the measurement of the construct and subsequent interpretation of results. Without them, we risk developing theorems that squander the full meaning of our findings [1].

Creativity is a complex construct which has been studied from a variety of perspectives [2–8]. This complexity has complicated the understanding of creativity–it is simply too multifaceted for a generalized model to be effectively applied. Of the countless definitions of creativity put forth in the literature [9, 10], two essential components–originality and effectiveness–have come to be widely accepted as essential to creativity [11, 12]. While this characterization is useful, and admittedly generalizable, its laxness permits substantial heterogeneity in scientific approach, as evidenced by the proliferation of ‘creativity tests’ which have been found to have limited theoretical correlation and validity [13–15]. Moreover, such a definition of creativity does not consider the contextual nature which dictates the originality and effectiveness of a given idea. For example, something novel and effective in the field of computer science may be ordinary and have little use in the field of psychology. Thus, a domain-specific approach is scientifically and practically important to understanding the process and products of creativity [16].

Surgery is a unique and safety-critical profession. This safety-critical nature is reflected in its standardized practices, protocols and training processes: through such standardization, variability in outcomes is reduced among surgeons and among patients, minimizing risk [17–20]. This standardization seems diametrically opposed to creativity, which has been associated with risk-taking [21–24]. Yet, creativity is useful, and often necessary, in high-risk, time-pressured situations, particularly when unforeseen problems arise: just consider the Apollo 13 mission, or the Mann-Gulch fire [25, 26]. Creativity in safety-critical situations can save lives–it follows that surgeons may also need the capacity to generate creative solutions to unforeseen issues.

Surgeons require a constellation of skills, including technical ability, communication, teamwork, decision-making, and leadership [27, 28]. Many of these skills are unique to the profession and likely interact with the necessary resources required for creativity–intelligence, knowledge, personality, motivation, and environmental factors–to constitute a surgeon’s creative potential [7]. If we are to validly measure the creative potential of surgeons and evaluate the products of creativity in the surgical context, a surgery-specific definition, reflecting the characteristics and competencies required for creativity in surgery as well as the contextual features of the profession, is essential. This definition will be particularly useful to creativity psychologists and surgical researchers looking to validly study the process of creativity in surgery and its implications for surgeons, their patients, and the healthcare system at large. With no such definition existing, we aim to generate a consensus definition of creativity in surgery using both the existing creativity literature and expert surgeon opinion.

## Methods and analysis

### Study purpose

The purpose of this study is to develop a consensus definition of creativity in surgery. The definition will provide the theoretical foundation from which creativity research in surgery can be conducted, facilitating its operationalization. In addition to developing a definition, we aim to characterize the creative surgeon and elucidate the relevance and importance of creativity in surgery.

### Study design

We plan to conduct a focus group, followed by a Delphi panel. Focus groups are a useful methodology for obtaining knowledge, perspectives, and explanations on a specific topic, and are particularly useful when existing knowledge is limited [29, 30]. Delphi studies use the Delphi method, which consists of iterative rounds of standardized surveys on a selection of anonymized panelists to achieve expert-based judgement on a given topic [31–33]. In addition to its use in the health sciences for developing standards or guidelines, measurement tools, and recommendations for action [32], the Delphi method has been utilized on several occasions to develop consensus definitions for terms lacking a clear or agreed-upon description [34–41]. Thus, the focus group and Delphi panel methodologies are suitable approaches for generating knowledge of creativity in surgery, which is limited, and developing a consensus definition of creativity in surgery.

### Steering group

A steering group will be constructed, consisting of at least 6 members. Each group member will possess extensive experience and training in health research methods, creativity research, statistics, and/or surgery. This multidisciplinary team will work together to conduct literature reviews, develop the Delphi content, and execute the study.

### Literature search

To identify the existing definitions of creativity in the literature, we will perform a systematic scoping review of the peer-reviewed literature on the MEDLINE, PsycINFO and EMBASE databases. We have developed a search strategy involving a combination of subject headings and search terms; the full search strategy is provided in S1 File. Title and abstract screening, full-text review, and data extraction will be performed independently and in duplicate, with discrepancies resolved by discussion until a consensus is reached. Upon completion of the full-text review, we will search the references of included papers for additional definitions.

### Expert focus group

We will begin by conducting a focus group comprised of highly experienced surgeons (10+ years of surgical experience), selected through non-probability purposive sampling of the chairs and/or chiefs of each surgical sub-specialty in the Department of Surgery at our home institution. We will seek to recruit between 4–12 focus group participants, in line with existing qualitative research guidance [42, 43]. Basic demographic characteristics (i.e., age; sex) and information on the focus group participants’ surgical experience (i.e., years of surgical experience; specialty) will be collected. The objective of the focus group will be to collect preliminary data on surgeons’ perceptions, attitudes and beliefs about creativity in surgery, collect real-world examples of creativity in surgery, and obtain expert opinions on the existing definitions of creativity in the literature. The data from the focus group will aid the construction of the initial definition statements used in the Delphi study. Given our objective of developing of definition generalizable to the entire domain of surgery, efforts will be made to construct a focus group from a range of surgical sub-specialties.

We developed a semi-structured topic guide to guide the focus group discussions. The guide will be integrated into Slido, a PowerPoint software add-on which facilitates live polling of audiences during presentations [44]. The following questions will be proposed to the focus group, to be rated via a 7-point Likert scale: ‘How important is creativity in surgery?’; ‘I have confidence in my ability to solve problems creatively’; ‘My work in surgery makes me more creative’; and ‘How important is surgical experience in the ability to be creative?’. Responses will be anonymized to reduce dominance and group conformity [31]. Once the focus group has submitted their responses, the results will be presented and used as a starting point for discussion, allowing the surgeons to elaborate on their responses.

We plan to explore the characteristics of a creative surgeon, posing the following question to the focus group: ‘What abilities or traits are required for a surgeon to be creative?’. The question will be an open text field, with multiple responses permitted. Once the focus group has submitted their responses, the results will be presented for further discussion. An open discussion on examples of creativity, inside and outside of the operating room, will also be had to collect real-world examples of creativity in surgery.

Using the existing literature, we identified 6 criteria which, in addition to novelty and effectiveness, have been considered key components of creativity: aesthetic, surprising, ethical, influential, authentic, and predictable [15, 45–49]. The focus group will be asked to select as many or none of the proposed criteria to add to the standard definition of creativity [11], to be considered as necessary components of creativity in surgery. A discussion period will follow to allow the focus group to expand on their responses.

Lastly, based on the results of our literature search, we will provide a list of existing definitions of creativity in the scientific literature, chosen by frequency of citation and relevancy to the profession as determined by the steering committee. The focus group will be asked to anonymously select the definition that is most relevant to the surgery. After all responses have been logged, the results will be presented and discussed.

### Analysis of focus group discussions

The responses to the Likert-measured questions will be descriptively analyzed and presented as medians, with interquartile ranges. Graphic representation of the data will be generated, where appropriate. Additionally, the discussions will be audio-recorded and transcribed verbatim. The primary researcher will perform a thematic analysis of the data, with the following process performed: active reading of the data; generation of the initial codes; theme identification; reviewing and categorization of themes; defining and finalizing themes; and reporting the findings [50]. We will perform the analysis utilizing a post-positivist approach, with the aim of maintaining the objectivity of the codes and minimizing researcher bias [51]. Reflexivity and establishment of structural coherence between the data and the interpretations will be practiced to maximize the trustworthiness of the results [52].

### Delphi design

Using the focus group data and definitions of creativity in the literature (chosen by frequency of citation, relevancy to the profession as determined by the steering committee, and variability among definitions), we will formulate five proposed definitions of creativity in surgery for use as initial Delphi statements. A statement formulation session will be held, led by the primary researcher with the support of the steering committee. Statements with greater abstractness and large amounts of information can impact the clarity of assessments, so all efforts will be made to ensure that statements are brief, utilize only the necessary amount of adjectives, and are easy to understand [53].

### Delphi participant recruitment

We will conduct non-probability purposive sampling of surgeons using email and in-person contact methods. Included surgeons will be licensed and practicing surgeons or surgeon trainees who have participated in surgical cases in the operating room. Members will be excluded if they cannot read, write or speak in English, are members of the steering committee, or were directly involved in the drafting of an existing creativity definition.

We will strive for a minimum panel size of 12 Delphi panellists, with no upper limit; research suggests that a minimum of 12 panellists are required to achieve a consensus [35]. Recruitment will begin in September 2024 and continue for a minimum of 2 months, or until the minimum sample size requirement is met.

### Delphi administration

The Delphi method will be conducted in the following fashion:

Round 1 –We will collect data on the panelists’ surgical specialty and years of surgical experience, as well as their perceived importance of creativity in surgery (7-point Likert) and its effect of the surgical profession on their creativity (7-point Likert). The panellists will then be presented with the five drafted definitions of creativity in surgery, choice-randomized, and asked to rank the definitions. An open-text field will allow panellists to suggest changes to the presented definitions.

Round 2–5 –If a consensus was not achieved after the first round, up to 5 rounds will be conducted. The suggested changes from the open-text field may be used to make alterations to the definitions. Following the presentation of the previous round’s results, the panellists will be asked to re-rank the definitions.

### Definition of consensus

The primary outcome of interest will be consensus on the definition of creativity in surgery. Research on Delphi studies has identified 75% agreement as the median threshold [54, 55]. This will be the *a priori* set threshold for this study. In the case that consensus on a definition is not reached, we will report the results of each round, explore reasons for the lack of consensus, and narratively discuss the implications of the findings on the understanding of creativity in surgery.

### Optimizing response rate

A large panel size and a high number of items are two factors significantly associated with a lower response rate [56]. Consequently, we will seek to recruit surgeons who have demonstrated interest in the research project and a willingness to participate in all rounds of the study. We will also ensure that each round takes no longer than 2 minutes to complete, and can be completed electronically on both computer and mobile platforms. The study team will personally conduct all recruitment of participants, as a ‘personal touch’ combined with a clear explanation of the importance and requirements of the study can help improve the response rate [57].

### Delphi response analysis

We will analyze the results of each Delphi round independently, performing descriptive analysis, reported as frequencies and percentages, to assess the responses of each round and determine the level of consensus on each item.

### Study schedule / timeline

Participant recruitment period is scheduled to begin on 1 August 2024 and continue until 1 November 2024. We expect data analysis and manuscript development to be complete, with a draft submitted for publication, by December 2024.

## Ethics and dissemination

### Ethical considerations

This proposed study has received ethics approval from the Hamilton Integrated Research Ethics Board (Project #16884). There is little risk in the participation of a Delphi study of this nature. Members of the focus group and Delphi panel will be required to sign an informed consent form to ensure they are aware of their rights and responsibilities in relation to the study.

### Knowledge translation

The research team has discussed the rationale and plan for the study with creativity psychologists, surgeons, and surgical researchers at previous conferences and in academic settings to understand the current methodological and theoretical issues we seek to address with this study. As such, we plan to publish the results of this study in a reputable surgical research journal or a creativity science research journal to reach the relevant stakeholders. We also plan to submit the results of this study for presentation at surgery and creativity science conferences locally, nationally, and internationally. Presenting at both surgical and creativity science conferences will provide the research team with feedback on the developed definition from a theoretical, methodological, and clinical perspective which will help guide future research efforts.

## Conclusion

Creativity is useful in safety-critical professions like surgery. Given the complexity and domain-specific nature of creativity, a surgery-specific definition is required to facilitate the valid scientific study of creativity in surgery. We plan to conduct a focus group and Delphi study to develop a consensus definition of creativity in surgery, utilizing surgeon opinion and guided by the existing scientific literature on creativity. The resulting definition will aid the valid scientific study of creativity in surgery by providing a conceptual foundation to guide the measurement and interpretation of surgical creativity research.

## Supporting information

S1 File Literature search strategies for MEDLINE, APA PsycINFO, and embase databases. (PDF)
