# Supplementary material for: A consensus definition of creativity in surgery: A Delphi study protocol
Source: PLoS One. 2024 Dec 5;19(12):e0314445. doi: 10.1371/journal.pone.0314445 (PMC11620345; doi:10.1371/journal.pone.0314445)
Supplement: S1 File — (PDF) [file pone.0314445.s001.pdf]

S1 File. Literature Search Strategies for MEDLINE, APA PsycINFO, and Embase databases.

APA PsycInfo

1	creativity/
2	(creativity or creative).ti,ab.
3	exp terminology/
4	Concept Formation/
5	(definition or define).ti,ab.
6	1 or 2
7	3 or 4 or 5
8	6 and 7

Embase

1	creativity/
2	(creativity or creative).ti,ab.
3	definition.ti,ab.
4	1 or 2
5	3 and 4

Ovid MEDLINE(R) and Epub Ahead of Print, In-Process, In-Data-Review & Other Non-Indexed Citations, Daily and Versions

1	Creativity/
2	(creativity or creative).ti,ab.
3	Terminology as Topic/
4	definition.ti,ab.
5	1 or 2
6	3 or 4
7	5 and 6
